# Effects of Parental Involvement in Robot-Assisted Autism Therapy

**DOI:** 10.1007/s10803-022-05429-x

**Published:** 2022-01-28

**Authors:** Aida Amirova, Nazerke Rakhymbayeva, Aida Zhanatkyzy, Zhansaule Telisheva, Anara Sandygulova

**Affiliations:** 1 Graduate School of Education, Nazarbayev University, Kabanbay Batyr Avenue, 53, Nur-Sultan, 010000 Kazakhstan; 2 Department of Robotics and Mechatronics, School of Engineering and Digital Sciences, Nazarbayev University, Kabanbay Batyr Avenue, 53, Nur-Sultan, 010000 Kazakhstan

**Keywords:** Robot-assisted therapy, Human–Robot interaction, Parental presence, Autism spectrum disorder, Attention deficit hyperactivity disorder

## Abstract

Parental involvement in traditional autism therapy is key to the effective treatment of children with ASD. Little is known about parental involvement in robot-assisted autism therapy (RAAT)—novel therapeutic support for children with ASD. Our study investigates the effect of parental presence on multiple-session RAAT conducted with 16 children with ASD. They interacted with the social robot in the presence or absence of their parents. We measured children's socio-behavioral outcomes and conducted semi-structured interviews with parents. Parents did not necessarily affect the children's outcomes during the interventions. However, children’s autism-related symptoms resulted in different socio-behavioral outcomes between sessions with and without parents. Most parents have reported positive changes in their children's behaviors when interacting with the robot.

## Introduction

Parents of children with Autism Spectrum Disorders (ASD) have long been involved in autism therapy. Their involvement can help better serve the special needs of children with ASD. Parental input to autism diagnosis and treatment can lead to effective treatment decisions and outcomes (Burrell & Borrego, 2012; Dimitrova et al., 2016). Evidence suggests that parent-mediated autism therapy can improve child development (Pickles et al., 2016; Schertz & Odom, 2007) and the quality of the child-parent relationships (McConachie & Diggle, 2006; Oono et al., 2013). In particular, children with ASD may benefit from parent-involved therapy in terms of communication skills (Coolican et al., 2010), social adaptivity (Beaudoin et al., 2019), and shared attention (Green et al., 2010).

In the current study, we take a closer look at how parents` involvement in a robot-assisted autism therapy (RAAT) affects children's engagement with the robot and overall socio-behavioral outcomes. Over the past two decades, RAAT has become an emerging field of research that traces social aspects of human–robot interaction (HRI) through therapeutic and pedagogical interventions with children with ASD. It can serve as a complementary therapy for children with autism and improve social communication skills since social robots can interact and engage with humans (Thill et al., 2013). They can retain simple and unnuanced social interaction and reduce the considerable workload of human therapists to focus on improving autism therapy (Andriella et al., 2018; Bharatharaj et al., 2017; Clabaugh et al., 2019). Likewise, Belpaeme et al. (2013) and Diehl et al. (2012) agreed that social robots might help children with socialization and motivation as well as with emotion recognition and empathy. We, therefore, remain enthusiastic about the future of robots for autism therapy. Indeed, robots can not replace humans but act as an assistant or mediator between children with ASD and co-present others, in our case, parents. Researchers in the HRI context usually rely on parent-reported data that are helpful to examine the robot’s effectiveness in assisting the children's social learning. To inform our research motivation, we first discuss how parents are represented in traditional and robot-assisted autism therapies.

## Literature Review

### Parents of Children with ASD

Parents of children with ASD have a complex parenting experience and develop a parent–child relationship not common to those with typically developing (TD) children. A possible explanation for this might be that the parents of children with ASD take on different learning, teaching, and caring roles that may be life-long (Autism Speaks, 2018). In this regard, parental self-efficacy and agency seem to be a significant concern (Crowell et al., 2019). For instance, numerous studies have reported parenting stress as a common experience for most parents raising a child with developmental disorders (Mcstay et al., 2013). In a longitudinal study with 54 families, Davis and Carter (2008) report that parents of toddlers deal with elevated levels of stress, acknowledging the results of prior studies with older children (Mcstay et al., 2013; Miranda et al., 2019). The study suggests that mothers experience more parenting stress than fathers because the mothers are the primary caregivers on many occasions. In another study, Guo et al. (2017) has found that dyadic interaction between a mother and a child with ASD had inconsistent emotion-engagement states (e.g. child negative/mother positive). In other words, children spend more time engaged with objects compared to their TD peers, who engage more with their parents. This finding reflects the past literature that describes children with ASD as attached to objects rather than humans. However, this view has been challenged by Richardson et al. (2018), which shows that autistic children can develop a relationship with people in their closest circle. In particular, emotional support and synchronisation between parents and children are efficacious in improving the overall social and emotional well-being (Crowell et al., 2019; Haven et al., 2014). Despite the challenges parents of children with ASD face, they remain an important source of socio-emotional support for their children.

### Parents in Traditional Autism Therapy

Parents play a crucial role in supporting their children's development in traditional autism therapy. Several researchers consider parents' role to be salient and undisputed in light of increased self-awareness of “autistic sociality'' among parents (Ochs & Solomon, 2010) and contribution to behavioural improvements in children (Diggle et al., 2003; McConachie & Diggle, 2006). Some studies take the further step to carefully look into how parental input at different stages of therapy could influence therapeutic outcomes. This input can help better serve the special needs of children with ASD. It has mainly taken on two forms. First, parental input in terms of diagnosis, planning, and treatment can lead to effective decisions made by specialists (Burrell & Borrego, 2012). Parents act as key informants on their children's well-being through interviews and conversations with therapists and researchers in this context. Parents` views and experiences have clinical relevance to help health professionals set up the therapeutic trajectory for children with ASD (Dimitrova et al., 2016; Jacobs et al., 2020). Second, parents may take on the role of the mediator between a therapist and a child in autism therapy. In a large-scale study, Green et al. (2010) tested the parent-mediated treatment with 152 children with core autism symptoms. Although the parent-mediators did not cause significant effects, the treatment helped parents encourage synchronous responses and shared attention in their children. In supporting parent-mediated interventions, Beaudoin et al. (2019) tend to improve parent–child engagement and behavioral outcomes such as social adaptivity of autistic children. Other positive examples of parental involvement include improved social communication (Pickles et al., 2016) and joint attention skills (Schertz & Odom, 2007). These results highlight parents' integral role in improving child outcomes in traditional autism treatment.

### Parents in Robot-Assisted Autism Therapy

While it has become clear that parental involvement in traditional autism therapy is widely accepted (Burrell & Borrego, 2012; Matson et al., 2009), a limited number of studies draw upon parental involvement in the RAAT context. Thill et al. (2013) are among them to emphasize that parental participation in early behavioral intervention could alleviate autism symptoms and achieve generalization. Yet, the current research on this topic is limited to parent-reported data collected from interviews and observations. That is because children with ASD may not reflect on therapy-brought improvements themselves. Therefore, it is necessary to collect parent observations of children's reactions to robots. For example, children with ASD preferred to be with caregivers during the intervention, and they tended to revert to close people when the novelty effect, caused by a robot, wore off (Richardson et al., 2018).

Despite having some concerns about technologies favored over traditional therapeutic interventions (Richardson et al., 2018), most parents remain optimistic about using social robots as aids in autism therapy. Remarkably, they tend to view robots as instructors rather than a companion or therapist (Oliver et al., 2019). They see a benefit in such interventions insofar as robots keep children socially engaged. The parents further note that a child may feel abandoned if the robot stops the therapy after being used for an extensive amount of time. In a different study, Berk-Smeekens et al. (2020) evaluated the adherence and acceptability of the robot-assisted PRT. Participants expressed positive views towards robots because of the increased enthusiasm and motivation they observed in their children (Berk-Smeekens et al., 2020). The authors emphasize the importance of parental involvement since it allows researchers to observe child behaviors outside the intervention and generalize improvements. Following this study, Otterdijk et al. (2020) reported that children remained attentive and engaged with their parents throughout the multiple robot-assisted PRT sessions. In general, parents seem supportive of social robots and their application in autism therapy.

### Current Study

Although parental involvement has been widely recognized in autism therapy, there is no direct evidence that parental involvement benefits children during robot-assisted ASD interventions. Our study seeks to investigate whether and how parental involvement in RAAT benefits children's socio-behavioral engagement. We take parental presence as a determining factor in children’s engagement during RAAT and focus on parent-driven effects on children's socio-emotional outcomes when interacting with a social robot. To this end, we hypothesize that parental involvement will affect children’s experience of robot-assisted autism therapy, and their autism-related and individual differences will lead to varying effects between two experimental conditions (i.e. the added value of the co-present parent compared to the no-parent condition). To the best of our knowledge, none of these research questions has been pursued across HRI studies, and research on parental involvement in ASD treatment remains limited.

## Methods

### Participants

16 children (n = 16) aged between 5 and 12 years old (M_age_ = 6.75 years old, SD = 2.14 years) diagnosed with ASD were recruited from a rehabilitation center in Nur-Sultan, Kazakhstan. Children’s Rehabilitation Center is a large hospital where children and their parents are admitted for 21 days to receive ASD treatment. Medical doctors conducted diagnostic tests, while two therapists observed each child’s differences on their first day in the center. Out of 16 children, eight were diagnosed with co-occurring ADHD.

The therapist performed an ADOS-2 (The Autism Diagnostic Observation Schedule-Second Edition) scoring test who has seven years of work experience and regularly conducts ADOS-2 at the Center. The mean score of the ADOS-2 test was 7.31 (SD = 1.54). Table 1 demonstrates children’s demographics such as age, data on their verbal skills, a co-occurring ADHD diagnosis, and ADOS-2 score provided by the therapist. It also includes the number of attended sessions and what sessions were attended with and without parents.

**Table 1:** Children’s characteristics and data on their sessions attended by each child with (P) and without parents (N)

Child	Age	Verbal	ADHD	ADOS-2	N	1	2	3	4	5	6	7	8	9	10
C1	5–6	–	–	8	6	P	N	N	P	N	P				
C2	5–6	–	–	5	6	P	N	P	N	N	N				
C3	8–9	–	–	8	6	P	N	N	N	N	N				
C4	5–6	✓	–	4	5	N	N		P	N	N				
C5	5–6	✓	✓	6	5	P	N	P		N	N				
C6	7–8	–	–	7	4		P	P		N	P				
C7	8–9	✓	–	9	2		P	N							
C8	10–11	✓	✓	9	9	P	P	N	N	N	N		N	N	N
C9	7–8	–	✓	9	9	P	N	P	P	P	P		P	P	P
C10	7–8	–	✓	8	8	P	P	P	P	N	N		N	N	
C11	5–6	–	✓	6	8	P	N	N	N		P	P	P		P
C12	5–6	–	✓	7	8		P	N	N		N	N	N	P	P
C13	6–7	–	–	8	7	P	P		P	P	P			N	P
C14	11–12	–	–	9	6	P	P	P	N					N	N
C15	9–10	✓	✓	8	6	P			N	N			N	N	N
C16	5–6	–	✓	6	7	P	P	P	N	N	P	P			

### Robot

This study used a child-sized NAO robot developed by Aldebaran Robotics in 2008 (Bertacchini, 2017). It is an autonomous and programmable robot successfully used as a companion for children, adults, and the elderly. It has basic modules such as built-in speech recognition, face recognition, display of gestures and body postures, and a text-to-speech engine that enables it to function more naturally and human-like (Amirova et al., 2021).

### Activities

Considering individual differences in behaviors between children with autism, we implemented multi-purposeful activities on the robot. They were predominantly based on the Applied Behavior Analysis (ABA) principles and targeted socio-emotional behaviors. To this end, we implemented 26 activities in two local languages (Kazakh and Russian) and categorized them into six groups (“Songs”, “Dances”, “Emotions”, “Social Acts”, “Imitations”, “Storytelling” and “Touch Me” (see Telisheva et al., 2019; Rakhymbayeva et al., 2021). Descriptions of each group are provided in Table 3. Although some activities overlap with each other in terms of targeted skills, each activity largely centers around one behavior (e.g. Emotions). Video demonstrations of the activities can be found at the link: bit.ly/rat-nu. The following ABA techniques were used throughout the interventions (Charlop-Christy et al., 2002; Chung et al., 2007; Leaf et al., 2017):

- *Positive reinforcement* was added throughout all robot activities to encourage children during the intervention. Children with autism need more stimulation and reward from the environment to practice behaviors and succeed in using them in the future. The robot was programmed to perform a number of verbal and non-verbal reinforcement behaviors. The examples of verbal praise include “Well done”, “Keep up the good work”, “Perfect”, and others. While non-verbal behaviors were the following: clapping hands, cheering, smiling, raising arms, and others. For instance, for the Touch me activity, we programmed the robot to clap hands accompanied by the corresponding sound when the child identified the correct body part and pressed the appropriate tactile sensor (e.g. left foot’s bumper). Additionally, at the end of each session, children were given a selection of stickers to choose from.
- *Picture exchange communication systems* (PECS) were utilized in several activities including Emotions, Transport, Animals and Storytelling. For example, for each emotion in the Emotions activity, the robot pointed at the photo with a situation (e.g. waiting on the bus stop), performed animation and told what emotion it felt (e.g. “I feel bored waiting for the bus”), and then pointed at the photo with a child showing that emotion (e.g. a boy being bored). Children’s joint attention was practiced in this activity. Similarly, the printed images of transports, animals, and storytelling characters were used as PECS in other activities.
- *Errorless teaching* is also applicable to our intervention framework. For example, in the “Touch Me” application, there was no differentiation between right and left arms and legs but only between arms, legs, and head tactile sensors. Also, when a child did not know how to touch the robot`s sensor correctly, a human therapist showed how to proceed.
- Focusing on *peer-mediated social skills training* (Battaglia & Radley, 2014), we programmed the robot’s role as a peer with the help of verbal utterances such as “let's play together”, “we are going to play”, and others. Additionally, the use of pronouns were included throughout all verbal utterances such as “we/ours” and informal “you/your” (Russian translation to “ты/твoй’’ and Kazakh translation to “ceн/ceнiң”). This is in fact demonstrated in the survey on the use of the NAO robot, i.e. children often refer to the NAO as a friend or a peer (Amirova et al., 2021).

### Setup

**Fig. 1:**
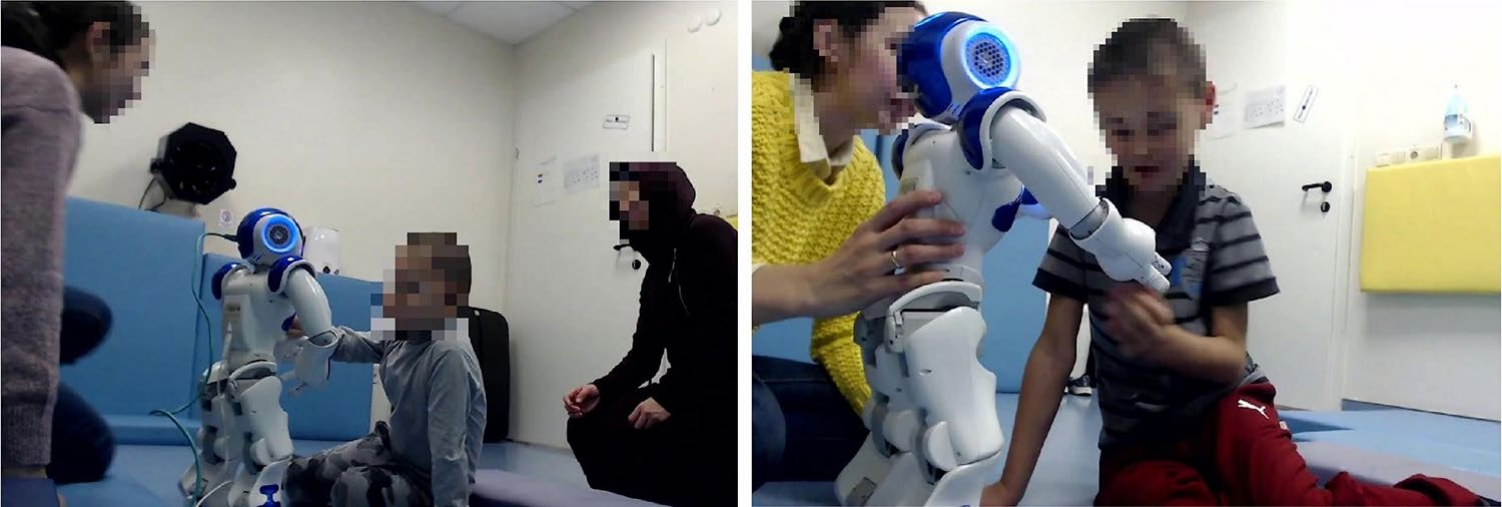
Experimental setup showcasing two conditions: with and without parents

The experiments were conducted on the premises of the rehabilitation center. They were carried out in a small room with only sport mats placed on the floor and walls. Two cameras, placed near a child and on the wall, recorded the sessions from two angles. To maintain eye contact with the robot, participants sat on the floor. Figure 1 displays the setup.

### The Role of the Therapist and Researchers

A therapist and one of the researchers were always in the room with a child for the therapist to administer the sessions and the order of the activities. In such a triadic setting, children interacted with the robot while the therapist coordinated their interaction. Additionally, the therapist was in charge of the child and robot safety. However, the researcher was sitting behind the folding screen hidden from the child view. As requested by the therapist, the researcher controlled the robot and launched the activities in a Wizard-of-Oz fashion via a Wi-Fi network.

#### Two Session Conditions: With and Without Parents

We distinguished two conditions to the type of intervention, with and without parents, by their physical presence in the intervention. We initially decided not to restrict parents with an assigned role during the intervention. They were given an opportunity to be present in the same environment as a child and the robot, and it was up to each parent to decide how to act. In most cases, parents provided prompts and repeated target behaviors with children or simply observed the intervention. In order to have two differentiated sessions, with and without parents, we asked parents to skip some sessions. Table 1 presents what sessions were attended with and without parents.

### Procedure

Each child could attend up to a maximum of 10 sessions. On average, each session lasted for 15 min, with the possibility to stop the session when a child has no interest to continue. Some children missed the sessions because of personal reasons (e.g. a child feeling unwell). It resulted in variations in the number of sessions between the participants, as presented in Table 1. The robot behaviors were introduced gradually depending on each child’s individual preferences and engagement with the robot. In the first session, the robot performed activities such as “Dances”, “Storytelling”, and “Touch Me”. Starting from the second session, the order of activities and the type of robot applications were tailored relying on individual engagement and liking. While some children did not prefer dance and song activities, others liked only them. We customized the order and type of activities based on the therapist’s observations and the parent’s feedback. After all sessions were completed, we interviewed parents and asked them about their children’s first reaction to the robot, observed changes in behavior, and overall reflection. In total, 10 out of 16 parents answered all of the questions consistently.

### Video Coding

All sessions were recorded by cameras with a built-in microphone. The cumulative number of hours of coded videos amounted to 25 h and 15 min. Once we video-coded the interactions, we calculated all 11 measures (see Table 2). Two researchers annotated 50% of the videos through the ELAN software. 20% of the data was cross-coded by another researcher. The inter-rater agreement on 20% of data was computed from pair-wise ICC of the coders and amounted to 82*.*6%. We used the coding strategy adopted in previous works on RAAT (see Table 2). Kim et al. (2012) coded fragments of videos that lasted for 10 s, while Rudovic et al. (2017) coded the whole engagement episode in the target task before one of the engagement scores was met. We coded engagement and valence scores relative to the timing of the activities. As such, each activity had several scores assigned to it (see Table 4 for a full list). Measures such as eye gaze and engagement duration were firstly counted and then calculated as percentages corresponding to the overall time of the session (e.g. engagement duration of 3 min out of 12-min session has a value of 25%). Additionally, we introduced a new measure "pressing the robot’s chest button". Although “button pressing” might be an example of repetitive behavior, the children in our study showed an act of naughtiness when they pressed it as it was not approved by the therapist (the robot would utter its IP address and distract the activity). Therefore, we coded and analyzed this measure separately from affective, curious, aggressive and stereotyped behaviors.

**Table 2:** Measures and their descriptions

Measures	Description	Adopted from
Engagement	Mean of engagement scores calculated for each session: a 5-point Likert scale, where 1—full non-compliance, 2—non-compliance, 3—several prompts, 4—one/two prompts, and 5—immediate reaction	Kim et al., (2012) Pop et al., (2014)
Valence	Mean of valence scores for each session: a 5-point scale: 1—cry/anger/fear, 2—sad/bored, 3—neutral, 4—interested, and 5—happy/excited	Kim et al., (2012) Rudovic et al., (2017)
Engagement time	The amount of time a child was engaged during one session. This variable is calculated relative to the overall time of the session (e.g. Engagement time is 25% i.e. 3 min out of 12 min-session)	Kim et al., (2012) Rudovic et al., (2017)
Eye gaze time	The amount of time a child spent looking at the robot calculated relative to the overall duration of the session	Admoni et al., (2011) Pop et al., (2014)
Affection	The duration of actions (kissing, hugging, tender touching, scratching, petting) of a child that are calculated relative to the overall duration of the session	Stanton et al., (2008)
Curiosity	The frequency of actions (opening, rotating, touching body parts) calculated relative to the overall duration of the session	Stanton et al., (2008) Rudovic et al., (2017)
Aggression	The frequency of actions (pushing, biting, hitting, pulling fingers) calculated relative to the overall duration of the session	Stanton et al., (2008) Pop et al., (2014)
Chest button	The total number of pressing a chest button for each session	
Stereotyped behaviours	The amount of time a child was flapping their hands, screaming, and crying were calculated relative to the overall time of the session	Stanton et al., (2008) Pop et al., (2014)
Smiles	The total number of times a child smiles in each session. Each second was coded as 1 for smiles and 0 if not detected	Pop et al., (2014) Rudovic et al., (2017)
Words	The total number of spoken words during a session linked to the time	Stanton et al., (2008)

#### Thematic Analysis of Parents’ Interviews

The semi-structured interviews were recorded in audio format. They lasted between 7 and 22 min. We used a six-step thematic analysis to process the interview data (Creswell, 2014; Miles et al., 2014). First, we prepared the data for analysis by transcribing the audio recordings into text format. To obtain a general sense of interviews, we read through the data. The further action was to organize and code the data by hand. Both deductive and inductive strategies were used to develop themes.

## Results

The analysis included 16 children (2 females) aged 5–12 years old who interacted with the robot either with or without parents. A series of Kolmogorov–Smirnov (K-S) and Shapiro–Wilk tests were conducted on all dependent variables overall and within groups to check the assumption of normality. Since all measures were normally distributed, a series of one-way repeated measures ANOVA and mixed ANOVA was used for the statistical data analysis presented in the following sections. We conducted Mauchly’s Test of Sphericity to check the assumption of sphericity. When it was violated, we used a Greenhouse–Geisser correction. We only report significant differences due to page constraints. Other results can be found in the Appendix in Tables 4–5.

### Parental Presence for All

First, we conducted a one-way repeated measures ANOVA with a Greenhouse–Geisser correction on a sample of 16 children to determine if there were differences in all measurements between sessions with and without parents. As Table 4 shows, there were no significant differences found between these two types of sessions (see Appendix A).

### Children with Severe ASD

**Fig. 2:**
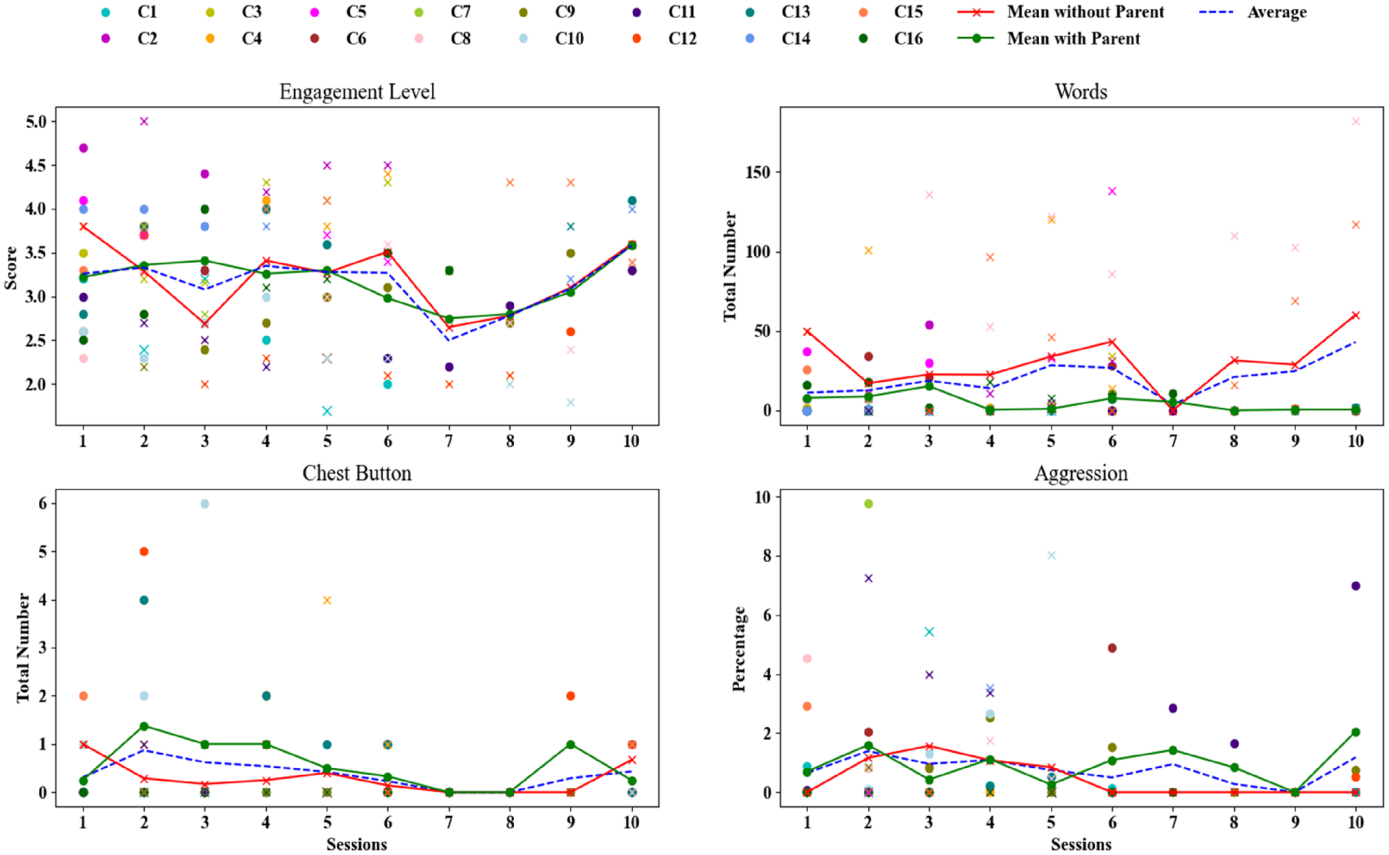
Measurements for each child for each session with significant results. Sessions with parents are labelled with a circle and sessions without parents are labelled with a cross (x) sign

**Fig. 3:**
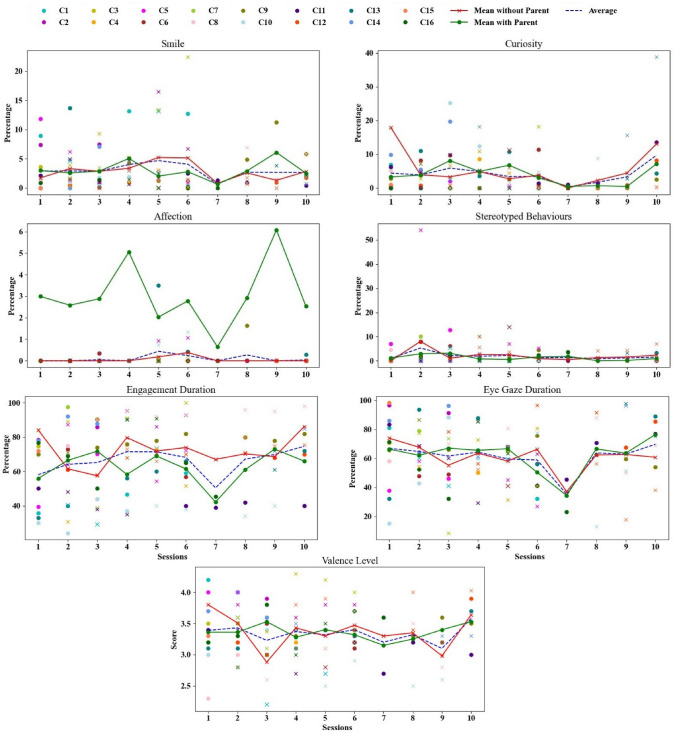
Measurements for each child at each session that did not produce significant results. Sessions with parents are labelled with a circle and sessions without parents are labelled with a cross (x) sign

We grouped the children by ADOS-2 score to understand if our hypothesis is accepted for children with severe autism with ADOS-2 score higher than 6 (N = 11). Similarly, we performed a separate analysis for children with moderate autism (N = 5, ADOS-2 < = 6). A one-way repeated-measures ANOVA with a Greenhouse–Geisser correction revealed that children with severe ASD pressed the chest button 0*.*803 ± 1*.*103 times on average during sessions with parents compared to only 0*.*085 ± 0*.*869 times during sessions without parents at the marginal level: *F*(1*,*10) = 4*.*963*, p* = 0*.*05. Figure 2 demonstrates these differences. Other measurements did not show significant differences (see Table 6 and Figure 3).

### ASD with Co-occurring ADHD

Then, we grouped children by their co-occurring ADHD diagnosis. As a result of the analysis, we had data of eight children diagnosed with ASD and eight children with a dual diagnosis of ASD and ADHD. Table 7 presents the results of a series of one-way repeated-measures ANOVA and mixed ANOVA tests showing non-significant differences in all 11 measurements between sessions with and without parents for these groups of children.

### Verbal and Non-verbal Children

Further, we grouped children into verbal and non-verbal categories. In total, 11 out of 16 children were non-verbal. A one-way repeated measures ANOVA statistical test showed that verbal children spoke fewer words during sessions with parents (15*.*4 ± 13*.*58) compared to those without (51*.*38 ± 29*.*35): *F*(1*,*4) = 7*.*952*, p* = 0*.*047. In contrast, non-verbal children scored higher engagement (3*.*318 ± 0*.*597) in sessions with parents as compared to sessions without them (2*.*966 ± 0*.*867): *F*(1*,*10) = 5*.*215*, p* = 0*.*045. In addition, we used a Mixed ANOVA test to compare the two types of sessions with the two groups of children (verbal and non-verbal). There are significant differences in the number of words spoken by verbal and non-verbal children during sessions with parents (15*.*4 ± 13*.*58 and 6*.*743 ± 11*.*560) as compared to sessions without parents (51*.*38 ± 29*.*35 and 3*.*384 ± 5*.*586): *F*(1*,*14) = 18*.*254*, p* = 0*.*0008. The frequency of aggressive actions was higher for verbal children (3*.*076 ± 4*.*005) than for non-verbal children (0*.*667 ± 0*.*945) during sessions with parents. By contrast, non-verbal children (0*.*985 ± 1*.*498) showed aggressive behaviour more often compared to verbal children (0*.*062 ± 0*.*138) during sessions without parents: *F*(1*,*14) = 6*.*595*, p* = 0*.*022. Figure 2 illustrates these differences, while Table 5 presents all the data for these groups.

### Interviews with Parents

*General impression of the robot-assisted therapy.* We first asked for information about parents’ thoughts regarding robot-assisted therapy and its overall effectiveness. Almost all parents portrayed it as a worthwhile experience that complements traditional therapies. They used the following phrases to describe their impression of the therapy: “very satisfied” (P2), “can’t criticize” (P3), “unique” (P4), “helpful” (P6, P8), and “novel and interesting” (P5, P10). Two parents shared their children’s impressions instead. While P1 said that his child was “less interested” in the robot as he has no specific interest in daily life. By contrast, P9’s child adored the robot and “looked for the robot every day”.

*Reaction to the robot.* Parents also shared their opinions on their children’s reaction to the robot, comparing it mostly with a toy or an animal. Overall, children’s reactions were rather positive as they showed an interest and curiosity towards the robot. However, P7 commented that her child was afraid of the robot: “She was frightened back then [during the first meeting] and still is”. Only a small number of parents considered the robot as “iron man” (P5) and “machine” (P8). A common view amongst parents (P1, P2, P9) was that their children reacted to the robot similar to a new unusual toy. P1 expressed that the first encounter might be compared to seeing a new thing or playing with a peer: “He was happy like when one sees a new thing. Also it was like he wanted to play with a little kid or a toy” (P1).

Aside from comparisons, some parents noticed a specific reaction in terms of behavior. Particularly, three parents observed that there was contact with the robot: “He came closer to it. He started to make contact and listened to it” (P3), and “He was attentive. He observed and looked [at the robot], which is rare with other people (P6). By contrast, one child remained quiet: “He was just lying there (smiles). He was calm at that moment” (P4).

*Behavioural changes.* It was really important to us to find out potential improvements in the behaviors of children with autism. Almost all parents reported some changes and improvements after the therapy. Parent-reported data provided us with remarkable insights into the positive changes in behaviors associated with the robot. However, P1 explained that it is difficult for his child to show immediate interest in everything, P4 was certain that therapy outcomes will be reflected on her child’s behaviors later on. Thus, it should be noted that children’s behavioral changes depend on their level of autism symptoms and individual differences. Most parents observed that their children wanted to speak more and pronounce new words. For instance, P3 noticed that her son really enjoyed the song’s activity and tried to sing: “Now he tries to sing a song. One day when we were in the canteen, he was trying to sing “chip-chip-chip” from the “Maria” song” (P3).

Importantly, one child could recognize his body parts and make eye contact according to P2: “He knows about heads. I think it is due to the robot that he can easily show where his head is. He knows about fingers. He also looks you in the eyes.” Likewise, P5 regarded eye contact as an improvement in her child: “I liked that he paid attention to the robot in the way that he was making eye contact.” Furthermore, P9 observed that her child performed movements: “He started doing body and arm movements. He used to sit before”. Similarly, P10 remembered that her child “danced happily to the Gangnam style song.”

More excitingly, P8 admitted that her child became curious about and attentive towards NAO, asking different questions about it: “He’s interested. He asks where the robot sleeps and if the second robot is [its] older or younger brother.” P6 highlighted the mediating role of the robot: “He opens up to other people through the robot. And I thought the robot would be a medium between him and other people”.

## Discussion

This study addressed the existing gap in autism research, which rarely studies the roles of family members in child-robot interaction (CRI). The current study is the first to explore the effect of parental presence on multi-session robot-assisted autism interventions. We considered parental presence one of the contributing factors that make the CRI effective.

### Parental Presence

Our hypothesis that parental involvement in autism interventions affects children’s interaction with a social robot revealed no significant differences in all 11 measures. Our general hypothesis is not supported. However, some interesting findings emerge when we account for the relationship between the heterogeneous nature of ASD and parental presence.

### Severe Autism

The severity level of autism was insignificant for how children engage with the robot. However, children with severe autism pressed the robot's chest button marginally more in parent sessions than in no-parent sessions. As the majority of these children were non-verbal, they might communicate with the robot through touch. This finding corroborates the results of past studies (Ferrari et al., 2009; Wainer et al., 2010), which showed that children might press buttons for sensory reward or communication. We consider that parental presence might trigger children to press the robot's chest button as an exploratory and playful behavior. That is, parental presence helps children with severe autism feel free-spirited and secure. This assumption challenges the results of past studies (Rutgers et al., 2004, 2007) where children with ASD were reported to be less securely attached to their parents or caregivers because of serious impairments such as severe autism and mental retardation. As their results were based on self-reported and cross-sectional data from relatively young groups of children, we believe that our finding offers a new perspective towards the impact of severe autism on the degree of parent–child bonding.

### Verbal Abilities

The verbal abilities of children with ASD were associated with different behavioral patterns. The verbal children with ASD showed aggressive behaviors and talked less in the presence of their parents than in their absence. This finding suggests that these children were more compliant to therapist’s and robot’s instructions in the absence of their parents, while the presence of parents made the children speak less, partially because parents spoke for them. Similarly, acting aggressively was more likely to happen in the presence of their parents due to the above mentioned non-compliance as well as feeling more confident next to their caregivers. Our findings suggest that parental involvement might not lead to expected positive gains for verbal children in our study since they have moderate to mild symptoms of autism that predict better communication skills to navigate social situations by themselves (Matson et al., 2008). By contrast, non-verbal children were more engaged, less aggressive, and spoke more in parent-involved sessions compared with no-parent sessions. This leads us to suggest that non-verbal children whose socio-behavioral skills are severely impaired benefit more from parental participation. This finding is comparable to that of Koegel et al. (2020), who suggested that verbal-focused treatments with parents are effective for non- or minimally-verbal children with ASD.

### Parent Observations

Interviews revealed that most parents observed positive and tangible changes in their children’s verbal and non-verbal behaviors after the intervention. Here, the most recurring observation was maintaining eye contact with the robot. Likewise, past research studies (Berk-Smeekens et al., 2020; Oliver et al., 2019) reported positive changes in verbal and non-verbal behaviors of their children with ASD after the robot-assisted therapy. Parents tend to perceive the robot as a toy, probably because of its small size. Our study has clarified that parents perceive social robots to have a therapeutic value, which is in line with previous literature (Berk-Smeekens et al., 2020; Butchart et al., 2021; Coeckelbergh et al., 2016). These reported data hold a significant value in identifying the overall efficacy of robot interventions for children with ASD. Besides, the parents' feedback will help us improve and design age-appropriate and cognitively demanding (e.g. educational) activities in the future.

Following these results, we refer to Burrell and Borrego Jr (2012) and Matson et al. (2009). They advocated for family involvement in ASD interventions because the child-parent relationship is a special bonding that influences social skills development. Parents’ roles and contributions are demonstrable and may yield significant improvements in behavioral development (Crowell et al., 2019; Haven et al., 2014; Thill et al., 2013). Parents and caregivers play an active role in documenting behavioral changes in children with ASD, and even in some cases, parents can initiate child-robot interactions (Pakkar et al., 2019; Scassellati et al., 2018). As Scassellati et al. (2018) noted, a social robot’s primary role is to improve the child-parent interaction – as children with ASD learn skills to direct to parents, not robots (as cited in Schembri, 2018). This statement highlights the robot’s role in reinforcing a human–human relationship. We suggest that parents make a big difference in autism therapy for children with severe impairments simply by being present in the same therapy room. For these children, interacting with a previously unseen robot may be a challenge that can be overcome with the help of parents or caregivers at the initial stages of the therapy.

## Key Takeaways on Parental Involvement in the RAAT

Our understanding of children with ASD and how to better address their needs has improved significantly. Beyond quantitative data and interviews, we believe our observations can also provide insightful ideas for those working with vulnerable populations in HRI settings. On the whole, most children seemed to feel relaxed due to their parents’ presence. Nevertheless, there are some exceptions to be considered in future studies. Some parents tend to control the child-robot interaction and insist on repeating the actions after the robot. For instance, one parent in our study pushed her child to interact with the robot in the first meeting. This action negatively impacted the attitude of the child towards the robot, who was unwilling and scared to interact with it in later sessions. This kind of help from parents seems ineffective because children can not learn new skills if parents intervene more often than expected. While most children (C1, C4, C5, C6, C7, C9, C10) were better engaged in the presence of their parents, some were more active (C2, C3, C8, C13, and C15) without their parents. Parenting style and child-parent relationship may substantially influence the intervention outcome, which needs further consideration.

Reflecting on our overall experience, there emerge some guidelines about how future studies could include parents in RAAT:

- (1) Parental input to the therapy helps identify behavioral characteristics and personal preferences of children with ASD if researchers consult with parents before conducting the interventions. The initial consultation with parents allows for the design of better treatment options.
- (2) Parents should be aware of their roles and responsibilities during therapy to minimize the unwarranted intervention.
- (3) For severe conditions of autism, parents could be present in the initial (few) sessions to allow their children to accustom to the new environment, robot and/or the therapist.
- (4) There might still be some children who need their parents around them at all times, and their whole experience with the robot would be impossible without their parents` presence.
- (5) Robots serve mediating roles and may stimulate special bonding between children and their parents or co-present others. Children tend to share their novel experiences with the robot with people closest to them.
- (6) Parents’ attitudes towards target activities may be collected through qualitative (e.g., interviews, observation) as well as quantitative (e.g. surveys) data collection methods. This would ensure the triangulation and transferability of results. Caregivers can identify and report minimal changes in their children’s behaviors and act as reliable informants. At the same time, their responses have to be interpreted with caution as there could be perception biases in parents.
- (7) Parents should be encouraged to take time for themselves. Some parents may stop attending the sessions after realizing that their children feel good and comfortable with the robot. They therefore can take that free time to recharge.
- (8) In collaboration with therapists, researchers may develop guidelines or tips for parents to use behavioral strategies such as positive reinforcement and prompting. These skills are helpful both within and outside therapy.

Measures/guidelines for parent-involved data collection:

1. Asking questions in the right place at the right time is demanding, but researchers could consider administering a pre-defined questionnaire to parents after each session. Our interview questions were general and less informative; future works should address this limitation. Interview questions should be aligned with the research questions of the study.
2. Exploring a parent–child relationship and parental self-efficacy before and after an intervention might be needed to account for their effect on the therapy. For instance, some use the Strange Situation Procedure (SSP) and Brief Attachment Screening Questionnaire (BASQ) to measure the parent–child attachment quality.
3. Using or establishing benchmarks for verbal and non-verbal skills that parents could report in easy to fill out forms to understand individual differences of children with ASD.
4. Administering pre- and post-tests to measure learning gains during the interventions as often performed in Robot-Assisted Learning applications with the robot. Cognitive engagement with the robot is one of the understudied research avenues in HRI.
5. Asking parents to keep a reflective diary to record any robot-related changes in their children’s behaviors (e.g. dancing like a robot, imitating sounds/movements taught by the robot, etc.). This is particularly helpful to document the dynamics of child-robot interaction.
6. Conducting follow-up interviews or delayed post-tests to understand long-term retention after RAAT interventions. These types of data collections could be performed via Zoom or WhatsApp calls.

We encourage researchers and medical specialists in autism therapy to make the best use of modern technologies in their practice. All of the participating children and their parents were mainly positive towards the robot. Its mediating role is an essential characteristic that results in an engaging experience and social stimuli. Involving parents is a step forward for child-robot interaction, creating an inclusive environment where parents’ roles are valued and acknowledged. More research is warranted in parental involvement.

## Conclusions

This long-term study investigated the effects of parental involvement on the social engagement of children aged 5–12 years old diagnosed with ASD. We evaluated children's socio-behavioral outcomes during the robot-assisted interventions with and without parents. Our results did not show the added value of parental involvement in robot-assisted therapy. However, the heterogeneous nature of ASD and parental involvement affected some intervention outcomes. Our results suggest that parent-involved RAAT benefits non-verbal children with severe impairments in social communication skills. They had better engagement when interacting with the robot in parent sessions than in no-parent sessions. Most parents in the study perceived the proposed RAAT as a worthwhile experience to support their children's social engagement. The implications of the study indicate parental involvement in robot-assisted ASD interventions as a key approach to supporting children with ASD. The major limitation of this study is the relatively small number of children. A further limitation is that the therapist's presence in both parent and no-parent sessions might also be regarded as a potential confounding factor. Future studies may replicate this study by expanding the number of participants and applying other target behaviors as well as changing interaction settings (e.g., home). We also encourage future research to explore how the child-parent relationship affects RAAT compared to traditional therapies, considering parenting styles and parent–child interaction quality. These factors may provide a better understanding of the robot-assisted autism treatment that puts the human relationship at the forefront of research. We believe that the child-parent relationship, mediated by a robot, is worth investigating in a technology-enhanced world.

## Data Availability

The dataset generated for this study will not be made publicly available because participants did not consent to future re-use of their video and interview data by other researchers. Deidentified individual participant data is available at the link: https://bit.ly/rat-data16.

## Appendix A

See Tables 3, 4, 5, 6, and 7.

**Table 3:** The description of robot activities

Group	Description
Dances	To maintain emotional well-being, children were encouraged to dance or listen to music along with the robot. This activity consists of off-the-shelf dances (“Gangnam style”, “Thai-Chi”, “Clapping” and “Macarena”). They were different in tempo and pace that enabled the range of the movements and music. Each dance is matched with music. The robot is activated by touching its tactile sensors on body parts
Songs	This activity focuses on songs with a similar choreography across the following activities: “Painter”, “Helper”, “Fixers”, “Wash your hands”, “Heroes”, “Clock”, “Spider”, “Mothers”, “Magdalena”, “Red Apricot”, “Tanya”, “Beautiful”). We created simple choreography using the audio files provided to us by the music therapist. They were also different in terms of tempo and pace. Song activities were programmed to be launched upon touching the robot’s tactile sensors on its head, left and right hands, and left and right feet
Emotions	This activity aims to exercise emotion recognition and joint attention. The robot displays five emotions (happy, sad, surprised, bored, and interested) using a matching sound such as a happy and crying sound, among others. Alongside the robot, there were printed emotion cards that depict a child expressing an emotional state. As an example, the robot says that a girl in the picture feels surprised when she sees rainbows. It then performs the emotion with interjections of surprise
Imitation	To encourage imitation skills, we created two activities known as “Transports” and “Animals”. The former activity comprises four means of transport (car, motorcycle, airplane, and boat) that were emulated by the robot. In the second activity, the robot demonstrates four animated behaviours of animals (gorilla, mouse, elephant, and horse). In the third activity, the robot demonstrates four animated behaviours of playing sport games (basketball, football, archery, and hockey). Each imitation was complemented with printed images and matching sounds. The robot stimulated children to imitate the movements and sounds
Touch Me	In this activity, children learn tactile contact with the robot. They also practice vocabulary related to body parts. The robot requests to touch one of its body parts, articulating common phrases, for example, “pat on my head”, “tap my blue toes on my right foot”, “stroke a blue spot on my right hand”, and so on. When a child touches the correct body part, the robot praises and applauds. It keeps quiet when a child touches the wrong body part
Social acts	This activity focuses on several behaviours to exercise social actions. It involves a set of non-verbal communication gestures such as hand clapping, peace sign, hugging, handshake, sending a kiss, high-five, and many others. These actions are accompanied by a statement (e.g. “I am yawning when I feel sleepy”, “I am clapping when I feel happy”). Following the robot’s demonstration, children are asked to repeat the action with their parents and/or with a therapist
Storytelling	To further support the emotional well-being of children, we introduced three storytelling activities, in which the robot acted out well-known fairy tales such as “The Bun”, “The Turnip”, and “The Cockerel”. The robot performs movements and sounds based on the storyline (e.g. pulling the turnip). Each story lasted for around 2–3 min

**Table 4:** Children's outcomes with and without parents

Measures	Sessions with parents Mean ± SD	Sessions without parents Mean ± SD	Parental factor one-way RM ANOVA
Smile	2.973 ± 3.130	3.106 ± 2.847	F(1,15) = 0.037, p = 0.851
Affection	0.075 ± 0.180	0.079 ± 0.204	F(1,15) = 0.003, p = 0.957
Aggression	1.42 ± 2.490	0.697 ± 1.303	F(1,15) = 1.053, p = 0.321
Curiosity	4.104 ± 4.187	4.931 ± 6.234	F(1,15) = 0.516, p = 0.484
Stereotyped behaviours	2.1 ± 3.403	3.262 ± 6.148	F(1,15) = 0.926, p = 0.351
Chest button	0.689 ± 1.022	0.225 ± 0.513	F(1,15) = 3.933, p = 0.066
Words	9.448 ± 12.467	18.384 ± 27.901	F(1,15) = 2.038, p = 0.174
Engagement	3.356 ± 0.552	3.125 ± 0.802	F(1,15) = 3.243, p = 0.092
Valence	3.325 ± 0.350	3.239 ± 0.373	F(1,15) = 0.571, p = 0.462
Eye gaze time	65.593 ± 16.818	64.014 ± 16.5	F(1,15) = 0.101, p = 0.755
Engagement time	65.796 ± 17.200	67.486 ± 18.628	F(1,15) = 0.102, p = 0.754

**Table 5:** The relationship between ADOS scores and parental presence

Measures	Sessions with Parents		Sessions without Parents		ADOS > 6 RM ANOVA for P vs N	ADOS < = 6 RM ANOVA for P vs N	Parent * ADOS Mixed ANOVA
	ADOS > 6 Mean ± SD	ADOS < = 6 Mean ± SD	ADOS > 6 Mean ± SD	ADOS < = 6 Mean ± SD			
Smile	2.765 ± 3.224	3.432 ± 3.069	3.195 ± 2.606	2.912 ± 3.221	F(1,10) = 0.258, p = 0.622	F(1,4) = 0.019, p = 0.897	F(1,14) = 0.384, p = 0.546
Affection	0.094 ± 0.213	0.034 ± 0.156	0.054 ± 0.299	0.134 ± 0.076	F(1,10) = 0.091, p = 0.769	F(1,4) = 2.635, p = 0.18	F(1,14) = 0.937, p = 0.349
Aggression	1.855 ± 2.866	0.462 ± 0.770	0.571 ± 2.178	0.974 ± 1.033	F(1,10) = 1.587, p = 0.236	F(1,4) = 0.048, p = 0.837	F(1,14) = 1.437, p = 0.251
Curiosity	4.647 ± 4.449	2.910 ± 7.130	5.746 ± 3.582	3.138 ± 3.7	F(1,10) = 1.018, p = 0.337	F(1,4) = 0.276, p = 0.627	F(1,14) = 0.116, p = 0.739
Stereotyped behaviours	1.971 ± 3.162	2.384 ± 4.064	2.722 ± 9.895	4.45 ± 4.278	F(1,10) = 0.087, p = 0.773	F(1,4) = 2.591, p = 0.183	F(1,14) = 0.242, p = 0.630
Chest button	0.803 ± 1.103	0.440 ± 0.152	0.085 ± 0.869	0.533 ± 0.876	F(1,10) = 4.963, p = 0.05	F(1,4) = 0.269, p = 0.631	F(1,14) = 2.906, p = 0.110
Words	6.689 ± 10.378	15.520 ± 25.286	12.339 ± 31.610	31.683 ± 15.706	F(1,10) = 0.0004, p = 0.985	F(1,4) = 2.601, p = 0.182	F(1,14) = 0.590, p = 0.455
Engagement	3.217 ± 0.432	3.662 ± 0.765	2.93 ± 0.783	3.554 ± 0.712	F(1,10) = 0.031, p = 0.864	F(1,4) = 6.753, p = 0.060	F(1,14) = 0.402, p = 0.536
Valence	3.364 ± 0.285	3.242 ± 0.399	3.168 ± 0.279	3.396 ± 0.496	F(1,10) = 3.068, p = 0.110	F(1,4) = 0.699, p = 0.450	F(1,14) = 2.170, p = 0.163
Eye gaze time	68.697 ± 14.395	58.766 ± 17.635	66.546 ± 13.675	58.442 ± 21.399	F(1,10) = 0.106, p = 0.751	F(1,4) = 0.766, p = 0.431	F(1,14) = 0.027, p = 0.871
Engagement time	66.950 ± 18.316	63.260 ± 18.871	66.637 ± 20.117	69.352 ± 16.096	F(1,10) = 0.0024, p = 0.962	F(1,4) = 0.017, p = 0.902	F(1,14) = 0.299, p = 0.593

**Table 6:** The relationship between autism diagnosis with co-occurring ADHD and parental presence

Measures	Sessions with Parents		Sessions without Parents		ASD with ADHD RM ANOVA for P vs N	ASD only RM ANOVA for P vs N	Parent * ADHD Mixed ANOVA
	ASD with ADHD Mean ± SD	ASD only Mean ± SD	ASD with ADHD Mean ± SD	ASD only Mean ± SD			
Smile	2.04 ± 2.04	3.99 ± 3.79	1.94 ± 1.05	4.28 ± 3.62	F(1,7) = 0.001, p = 0.972	F(1,7) = 0.053, p = 0.823	F(1,14) = 0.048, p = 0.829
Affection	0.05 ± 0.09	0.10 ± 0.25	0.15 ± 0.28	0.01 ± 0.03	F(1,7) = 1.189, p = 0.311	F(1,7) = 1.121, p = 0.324	F(1,14) = 2.311, p = 0.151
Aggression	1.22 ± 1.23	1.62 ± 3.42	1.02 ± 1.7	0.37 ± 0.71	F(1,7) = 0.106, p = 0.753	F(1,7) = 0.928, p = 0.367	F(1,14) = 0.531, p = 0.478
Curiosity	3.27 ± 3.84	4.94 ± 4.61	2.83 ± 2.91	7.04 ± 8.04	F(1,7) = 0.106, p = 0.754	F(1,7) = 1.313, p = 0.289	F(1,14) = 1.240, p = 0.284
Stereotyped behaviours	2.02 ± 3.30	2.11 ± 3.73	4.02 ± 7.53	2.51 ± 4.79	F(1,7) = 1.310, p = 0.289	F(1,7) = 0.048, p = 0.832	F(1,14) = 0.386, p = 0.545
Chest button	0.92 ± 1.23	0.46 ± 0.78	0.16 ± 0.25	0.26 ± 0.61	F(1,7) = 3.193, p = 0.117	F(1,7) = 1.000, p = 0.350	F(1,14) = 1.692, p = 0.214
Words	10.07 ± 13.11	8.88 ± 12.68	24.83 ± 31.18	11.94 ± 24.51	F(1,7) = 3.920, p = 0.088	F(1,7) = 0.091, p = 0.770	F(1,14) = 0.874, p = 0.366
Engagement	3.06 ± 0.36	3.64 ± 0.59	3.35 ± 1.35	3.41 ± 0.80	F(1,7) = 1.345, p = 0.284	F(1,7) = 1.788, p = 0.222	F(1,14) = 0.006, p = 0.941
Valence	3.27 ± 0.32	3.50 ± 0.17	3.19 ± 0.37	3.29 ± 0.39	F(1,7) = 0.039, p = 0.847	F(1,7) = 2.063, p = 0.194	F(1,14) = 1.138, p = 0.304
Eye gaze time	62.46 ± 17.24	69.74 ± 15.43	62.28 ± 15.80	65.75 ± 18.08	F(1,7) = 0.008, p = 0.929	F(1,7) = 0.647, p = 0.447	F(1,14) = 0.222, p = 0.645
Engagement time	56.98 ± 13.43	73.88 ± 16.36	68.26 ± 20.41	66.71 ± 18.05	F(1,7) = 3.220, p = 0.115	F(1,7) = 0.805, p = 0.399	F(1,14) = 3.192, p = 0.096

**Table 7:** Verbal outcomes with and without parents

Measures	Sessions with Parents		Sessions without Parents		Verbal RM ANOVA for P vs N	Non-Verbal RM ANOVA for P vs N	Parent * Verbal Mixed ANOVA
	Verbal Mean ± SD	Non-Verbal Mean ± SD	Verbal Mean ± SD	Non-Verbal Mean ± SD			
Smile	1.946 ± 2.554	3.440 ± 3.364	2.640 ± 1.119	3.318 ± 3.390	F(1,4) = 0.391, p = 0.565	F(1,10) = 0.018, p = 0.895	F(1,14) = 0.281, p = 0.604
Affection	0.034 ± 0.076	0.093 ± 0.212	0.134 ± 0.299	0.053 ± 0.156	F(1,4) = 1.000, p = 0.373	F(1,10) = 0.218, p = 0.650	F(1,14) = 0.937, p = 0.349
Aggression	3.076 ± 4.005	0.667 ± 0.945	0.062 ± 0.138	0.985 ± 1.498	F(1,4) = 2.816, p = 0.168	F(1,10) = 0.674, p = 0.430	F(1,14) = 6.595, p = 0.022
Curiosity	2.800 ± 3.344	4.697 ± 4.538	2.062 ± 3.054	6.235 ± 6.970	F(1,4) = 1.539, p = 0.282	F(1,10) = 0.887, p = 0.368	F(1,14) = 0.830, p = 0.378
Stereotyped behaviours	4.530 ± 5.099	0.995 ± 1.637	5.482 ± 9.551	2.252 ± 4.077	F(1,4) = 0.065, p = 0.811	F(1,10) = 2.443, p = 0.149	F(1,14) = 0.013, p = 0.911
Chest button	0.800 ± 1.095	0.639 ± 1.038	0.440 ± 0.876	0.127 ± 0.231	F(1,4) = 1.000, p = 0.373	F(1,10) = 2.747, p = 0.128	F(1,14) = 0.085, p = 0.775
Words	15.400 ± 13.580	6.743 ± 11.560	51.383 ± 29.348	3.384 ± 5.586	F(1,4) = 7.952, p = 0.047	F(1,10) = 1.505, p = 0.247	F(1,14) = 18.254, p = 0.0008
Engagement	3.440 ± 0.487	3.318 ± 0.597	3.474 ± 0.552	2.966 ± 0.867	F(1,4) = 0.027, p = 0.875	F(1,10) = 5.215, p = 0.045	F(1,14) = 2.079, p = 0.171
Valence	3.280 ± 0.410	3.346 ± 0.340	3.408 ± 0.258	3.162 ± 0.400	F(1,4) = 0.472, p = 0.529	F(1,10) = 1.766, p = 0.213	F(1,14) = 1.673, p = 0.217
Eye gaze time	64.960 ± 23.199	65.881 ± 14.446	57.148 ± 14.404	67.134 ± 17.061	F(1,4) = 0.461, p = 0.534	F(1,10) = 0.057, p = 0.815	F(1,14) = 0.701, p = 0.417
Engagement time	70.830 ± 17.860	63.509 ± 17.256	67.610 ± 20.284	67.429 ± 18.866	F(1,4) = 0.037, p = 0.855	F(1,10) = 1.646, p = 0.228	F(1,14) = 0.374, p = 0.551

## Appendix B

See Fig. 3.
